# Enhancing dendritic cell immunotherapy for melanoma using a simple mathematical model

**DOI:** 10.1186/s12976-015-0007-0

**Published:** 2015-06-09

**Authors:** E. Castillo-Montiel, J. C. Chimal-Eguía, J. Ignacio Tello, G. Piñon-Zaráte, M. Herrera-Enríquez, AE. Castell-Rodríguez

**Affiliations:** Laboratorio de Modelación y Simulación, Centro de Investigación en Computación, Instituto Politécnico Nacional, Av. Juan de Dios Bátiz, Esq. Miguel Othón de Mendizábal, Del. Gustavo A. Madero, México City, 07738 México; Departamento de Matemática Aplicada a las Tecnologías de la Información y las Telecomunicaciones, E.T.S.I. Sistemas Informáticos, Universidad Politécnica de Madrid, Ctral. de Valencia. Km. 7, Madrid, 28031 Spain; Laboratorio de Inmunoterapia e Ingeniería de Tejidos, Departamento de Biológia Celular y Tisular, Facultad de Medicina, Universidad Nacional Autónoma de México, Edificio A, Sexto Piso, Ciudad Universitaria, Av. Universidad No. 3000, México City, 04510 México

**Keywords:** Mathematical model, Cancer, Melanoma, Immunotherapy, Dendritic cell, *TGF*−*β* cytokine

## Abstract

**Background:**

The immunotherapy using dendritic cells (DCs) against different varieties of cancer is an approach that has been previously explored which induces a specific immune response. This work presents a mathematical model of DCs immunotherapy for melanoma in mice based on work by Experimental Immunotherapy Laboratory of the Medicine Faculty in the Universidad Autonoma de Mexico (UNAM).

**Method:**

The model is a five delay differential equation (DDEs) which represents a simplified view of the immunotherapy mechanisms. The mathematical model takes into account the interactions between tumor cells, dendritic cells, naive cytotoxic T lymphocytes cells (inactivated cytotoxic cells), effector cells (cytotoxic T activated cytotoxic cells) and transforming growth factor *β* cytokine (*TGF*−*β*). The model is validated comparing the computer simulation results with biological trial results of the immunotherapy developed by the research group of UNAM.

**Results:**

The results of the growth of tumor cells obtained by the control immunotherapy simulation show a similar amount of tumor cell population than the biological data of the control immunotherapy. Moreover, comparing the increase of tumor cells obtained from the immunotherapy simulation and the biological data of the immunotherapy applied by the UNAM researchers obtained errors of approximately 10 *%*. This allowed us to use the model as a framework to test hypothetical treatments. The numerical simulations suggest that by using more doses of DCs and changing the infusion time, the tumor growth decays compared with the current immunotherapy. In addition, a local sensitivity analysis is performed; the results show that the delay in time “ *τ*”, the maximal growth rate of tumor “r” and the maximal efficiency of tumor cytotoxic cells rate “*aT*” are the most sensitive model parameters.

**Conclusion:**

By using this mathematical model it is possible to simulate the growth of the tumor cells with or without immunotherapy using the infusion protocol of the UNAM researchers, to obtain a good approximation of the biological trials data.

It is worth mentioning that by manipulating the different parameters of the model the effectiveness of the immunotherapy may increase. This last suggests that different protocols could be implemented by the Immunotherapy Laboratory of UNAM in order to improve their results.

**Electronic supplementary material:**

The online version of this article (doi:10.1186/s12976-015-0007-0) contains supplementary material, which is available to authorized users.

## Background

Melanoma is a dangerous form of skin cancer and its prevalence is increasing at a dramatic rate worldwide [1, 2]. In the last decades, there have been significant advances in the treatments for early stage melanoma with a high survival rate but not for the latter invasive stage where treatments are limited [3, 4]. One of these treatments is the immunotherapy for melanoma which activates the immune response and stimulates the mechanisms of defence against cancer [3, 5, 6].

The immunotherapy used the tumor cell property to express antigens than can be recognized by the immune system and became targets of the tumor-specific T cells [4, 7, 8]. This is to stimulate and boost the immune response to tumor-specific cells and not to injure the normal cells using tumor-specific antigens, mature dendritic cells (DCs), T-cells or cytokines [5, 9]. Lately, the identification of tumor antigens and the advance in the understanding of the immune system has allowed the development of new immunotherapies [5].

The antigen-specific immunotherapy with DCs uses the capacity of present antigens and activates the immune specific response of DCs. Immature DCs in a steady state located in epithelial and connective tissues have the ability to detect and capture antigens that are found. After capturing the tumor antigens, these mature and transport the antigen to lymph nodes where they present the tumor antigen to naive T lymphocyte cells (*CD*4^+^ and *CD*8^+^ T lymphocytes). These encounters activated *CD*8^+^ lymphocytes proliferating and differentiating into CTLs leaving the lymphoid organs where they were generated and migrate to eliminate the tumor cells [5, 10].

Tumor cells use the mechanisms of host defense to promote tumor progression, invasion, and dissemination to distant sites. One of these mechanisms is by secreting *TGF*−*β* cytokine which damped the immune response. For example, interfering with the antigen transportation to lymph nodes or affecting the *CD*4^+^ and *CD*8^+^ effector functions (proliferation, differentiation, and acquisition of effector molecules) [11, 12].

In this immunotherapy, the DCs taken from the patient were incubated with tumor antigens and adjuvants *in vitro* and then injected back into the patient. The immunotherapy is specific because it only eliminates tumor cells and does not injure most normal cells in patient [5].

There is hope that one of these immunotherapies, the Sipuleucel-T treatment shows that the personalized treatment with antigen-presenting cells (APCs) could be efficient to extend the life of people suffering from prostate cancer by 31.7 *%* over a 36 month period [13]. For that reason the investigation with dendritic cells is one of the immunotherapy treatments being studied and improved lately. The research group at Medicine Faculty of UNAM are developing a immunotherapy using DCs infusion on mice with melanoma cancer and have up till now prolonged their life by 33 *%* over 34 days [14].

The UNAM researchers incubate the DCs derived from mice bone marrow with granulocyte-macrophage colony stimulating factor (GM-CFS) to mature the cells and antigen *MAGE*−*AX* peptide which stimulate the immune response before being injected into the mice. The biological treatment efficiency is measured taking into consideration the tumor diameters, cytokines modulation (IL-2 and IL-10), the expression of major histocompatibility complex molecules (MHC) and the survival of the mice.

However, the tumor has not been eradicated and there are still many unanswered questions about how the immune system interacts with the tumor cells, and which components of the immune system play significant roles in responding to immunotherapy. Actually, they used only one infusion protocol (see Table 1 Protocol 1). This is applied in all their immune treatments and they are looking for a new infusion protocol to improve their results.

**Table 1:** Immunotherapy protocol of dendritic cells (DCs)

Infusion time					
Protocol	week 1	week 2	week 3	week 4	week 5
	(0 hrs)	(168 hrs)	(336 hrs)	(504 hrs)	(672 hrs)
Control mice	Infusion of				Mice
	6×10^4^				sacrifice
	B16/F10 cells				
Protocol 1	Infusion of	Infusion of	Infusion of	Infusion of	Mice
(Treatment of DCs with MAGE)	6×10^4^	10^6^ DC	10^6^ DC	10^6^ DC	sacrifice
	B16/F10 cells				

In this way mathematical models may provide an analytic framework to address questions and these models can be used both descriptively and predictively for the new therapies [15, 16]. An example of the success of this type of mathematical models was performed by Kronik et. al. [17], they developed a personalized mathematical model to simulate the interaction between allogenic prostate cancer (PCa) whole-cell vaccine and the immune system in patient. They validate their results with clinical trials tests.

This differs from our work in the sense that we propose a mathematical model Ad hoc of the immunotherapy developed by the research group at Medicine Faculty of UNAM. The consequences of manipulating some of the parameters associated with a particular treatment is explored; also, this model is used to study hypothetical immunotherapy protocols and examines the consequences in the growth of the tumor cells population.

## Results

### Simulation and validation of tumor cells growth

To simulate the tumor cells growth without immunotherapy an initial population of 6∗10^4^ tumor cells to induce melanoma in mice is taken (Table 1) and the parameters listed in the additional material (see Additional file 1) with the parameter of maximal efficiency of cytotoxic cells set to 0, i.e. “ *a*_*T*_=0”.

Normalized root mean square error (NRSME) between the real data and the numerical simulation is obtained, taking into account the measurement time made by the UNAM researchers (the 7th day after melanoma cell induction and every two days after the 10th day for a period of 700 hours). The population of the tumor cells in the melanoma tumor was calculated from the diameters average of tumor melanoma assuming a spheric form (size of tumor cells equal to 17.4±0.21*μ* m [18]).

**Fig. 1:**
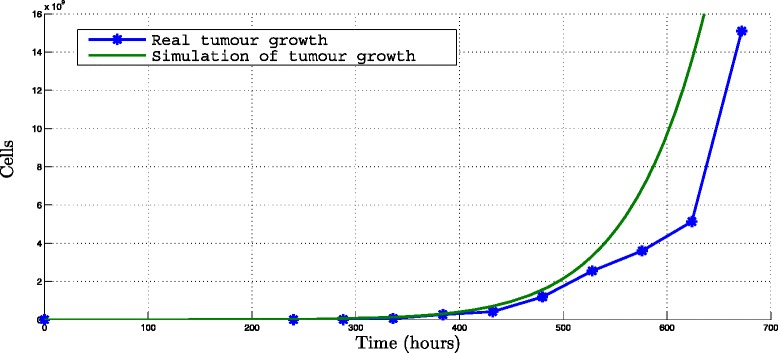
Simulation of tumor growth. Simulation for 700 hours of control mice from *T*(0)=6×10^4^, *r*=0.00106 *h* ^−1^ and *K*=6.754^15^ *c* *e* *l* *l* *s* (Table 1)

Figure 1, show the data of real tumor cellular growth and the tumor cellular growth obtained by the mathematical model. It can be seen that the growth of the tumor is interrupted at 672 hours because all the surviving mice were sacrificed after the fourth week.

**Fig. 2:**
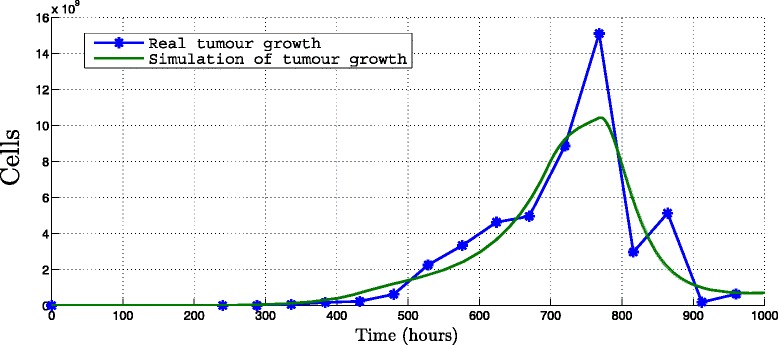
Simulation of immunotherapy. Simulation applying the immunotheraphy Protocol 1 for 1000 hours, set to *T*(0)=6×10^4^, *r*=0.00106*h* ^−1^ and *K*=6.754^15^ *c* *e* *l* *l* *s* (Table 1)

Given the immunotherapy protocol of the UNAM researchers which administered three doses of 10^6^ DCs activated with MAGE-AX, infused every 168 hours during three weeks (Table 1, Protocol 1) and with the parameters listed in additional material (see Additional file 1), the simulation of immunotherapy is realized. In the same manner as NRSME is calculated to measure the difference of the tumor growth without immunotherapy we obtain a 10.5 *%* NRMSE between the real population of tumor cells and the simulated population of tumor cells applying immunotherapy Protocol 1 described in Table 1 (see Fig. 2).

### Proposed immunotherapy protocols

Considering the immunotherapy protocol used by the UNAM researchers could be improve, we suggest different immunotherapy protocols to eradicate the tumor and to extend the life of mice.

#### Modification of the amount injected DCs

To test different quantities of injected DCs, we use the same protocol of injection of DCs in the biologic treatments (three doses each 168 hours in 1000 hours) and we change the amount of DCs injected per dose. The numerical results are shown in Table 2 (from Protocol 2 to 9).

**Table 2:** Hypothetical immunotherapy protocol simulated

Protocol	DC	Inj. Interval	Number of	% of DCs	% of Decrease	Erad
	doses	(hours)	doses			
2	10^2^	168	3	50	12.61	*no*
3	10^3^	168	3	50	19.92	*no*
4	10^4^	168	3	50	20.79	*no*
5	10^5^	168	3	50	20.95	*no*
6	10^6^	168	3	50	68.65	*no*
7	10^7^	168	3	50	88.69	*yes*
8	10^8^	168	3	50	91.68	*yes*
9	10^9^	168	3	50	91.68	*yes*
10	10^6^	48	8	50	85.97	*yes*
11	10^6^	72	5	50	83.70	*yes*
12	10^6^	96	4	50	81.40	*yes*
13	10^6^	120	3	50	76.05	*no*
14	10^6^	144	3	50	76.72	*no*
15	10^6^	192	2	50	37.12	*no*
16	10^6^	216	2	50	33.11	*no*
17	10^6^	168	3	100	80	*yes*
18	10^6^	168	3	80	77.58	*yes*
19	10^6^	168	3	60	73.05	*no*
20	10^6^	168	3	40	60.33	*no*
21	10^6^	168	3	20	22.16	*no*

*Inj. Interval* Injection Interval, *Erad* Eradication, *%* of DCs Percentage of DCs that arrive at the beginning of the immune response.

To measure the efficiency of the proposed protocol, the average reduction of tumor cells (in percentage terms) is computed comparing the tumor cell population simulated without treatment and the tumor cell population simulated with the hypothetical treatment measured at the same time (the 7th day after beginning the simulation and every two days after the 10th day for a period of 1000 hours).

**Fig. 3:**
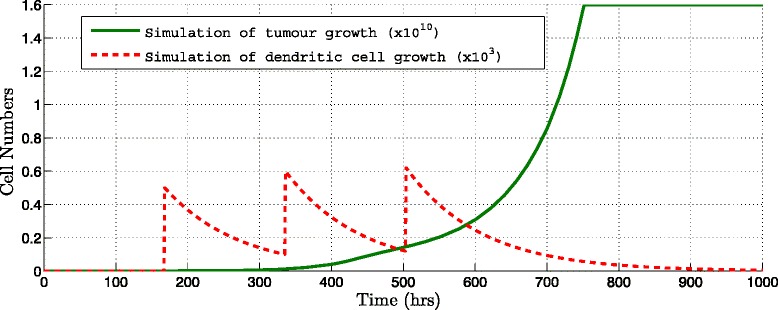
Simulation of immunotherapy for different amounts of DCs per dose. Interval injection every 168 hours during three weeks and applying 10^3^ DCs per dose (Table 2, Protocol 3), a 19.92 *%* of decrease was obtained. Tumor population (*thick line*) and amount of DCs injected (*dotted line*) are shown

**Fig. 4:**
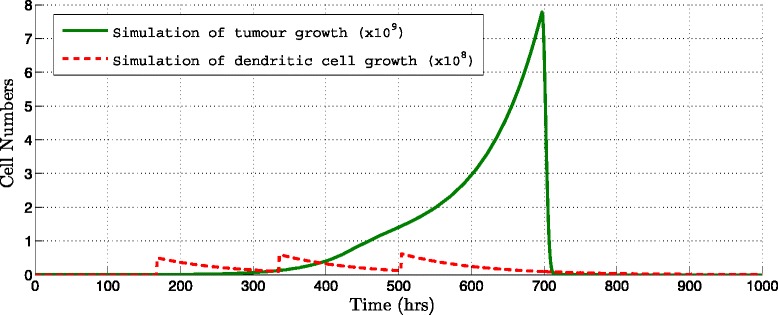
Simulation of immunotherapy for different amounts of DCs per dose. Interval injection every 168 hours during three weeks and applying 10^8^ DCs per dose (Table 2, Protocol 8) with a 88.69 *%* of decrease calculated. Tumor population (*thick line*) and amount of DCs injected (*dotted line*) are shown

Figures 3 and 4 show some of the simulation results from immunotherapy protocol proposals where the amount of DCs injected is changed. Figure 3, shows numerical results considering injections of 1∗10^3^ DCs every 7 days during 3 weeks (dotted line) it can be observed that the growth of the tumor does not suffer any change and continues growing, demonstrating that this amount is not sufficient to eliminate or to detain the growth of the tumor (thick line) in the simulation.

On the other hand, Fig. 4 shows the results of the 1∗10^8^ DCs injected every 7 days during 3 weeks; the simulation shows how the tumor cells population declines dramatically after 700 hours.

#### Modification of the immunotherapy interval

It is proposed to modify the infusion interval time of the DCs, conserving the same number of DCs used in the biological experiment (10^6^ DCs per dose, Protocol 1), to show the influence of the number of infusions in the treatment. As in the other experiments, the efficiency of the hypothetical protocol is measured using the average reduction of tumor cells. The numerical results are shown in Table 2 (from Protocol 10 to 16).

From the results shown in Table 2 it can be observed that the tumor is eradicated for intervals of injection less than 120 hours in a range of 1000 hours of simulation.

**Fig. 5:**
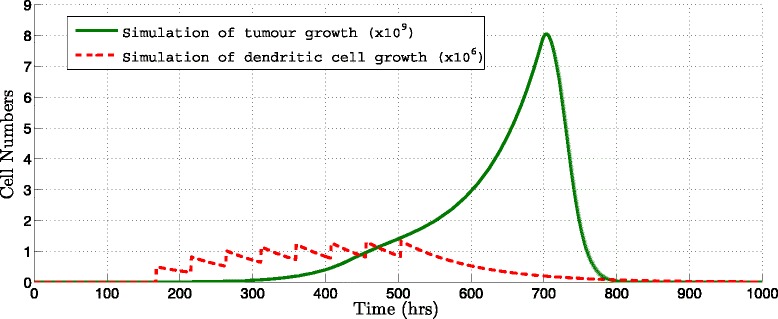
Simulation of immunotherapy changing the number of DCs doses. Interval injection every 48 hours applying 10^6^ DCs per dose during three weeks (Table 2, Protocol 10) a 85.97 *%* of decrease was obtained. Tumor population (*thick line*) and amount of DCs injected (*dotted line*) are shown

**Fig. 6:**
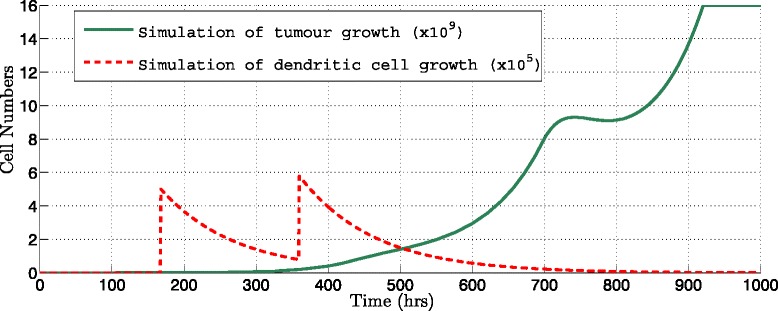
Simulation of immunotherapy changing the number of DCs doses. Interval injection every 192 hours applying 10^6^ DCs per dose during three weeks (Table 2, Protocol 15) a 37.12 *%* of decrease was obtained. Tumor population (*thick line*) and amount of DCs injected (*dotted line*) are shown

Figures 5 and 6, show how the immunotherapy is more efficient if the interval of injection is reduced and the number of injections increased. Applying only one more dose and reducing the injection interval of 168 hours to 120 hours the simulation shows that the tumor is eradicated.

#### Modification of DCs doses and the applied immunotherapy interval

Doses of 10^2^,10^3^,10^4^,10^5^,10^6^,10^7^,10^8^ and 10^9^ DCs were tested with injected intervals of 48, 72, 96, 120, 144, 168 and 192 hours. The results indicate that the tumor is eradicated for all protocols of DCs doses bigger than 10^7^.

Using doses of 10^6^ DCs with intervals less than 120 hours in a time period of 1000 hours, the tumor is eradicated. Nevertheless, for intervals of injection longer than 120 hours, the tumor cells survive after 1000 hours. In the case where the doses are less than 10^6^ DCs, the tumor cells population continued growing.

#### Percentage of DCs that induce the immune response

To find out the importance of the percentage of DCs that reach the lymph nodes, the immunotherapy protocol used by the research group of UNAM is simulated changing the percentage of DCs injected per doses.

The numerical simulation show that if the percentage of DCs arriving at the lymph nodes increases up to 80 *%*, the tumor is eradicated (see Table 2 from Protocol 17 to 21).

#### *TGF*−*β* cytokine importance

**Fig. 7:**
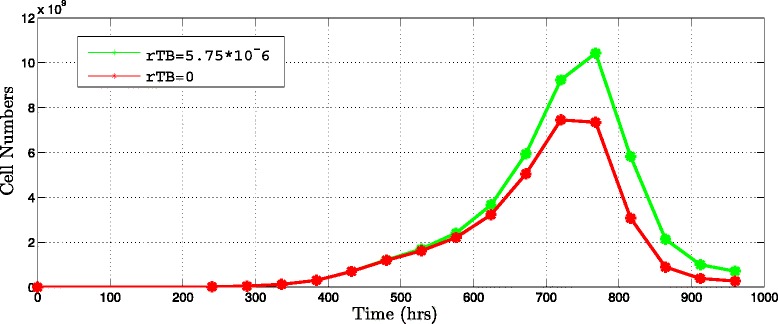
Effect of the *T* *G* *F*−*β* cytokine in the immunotherapy. Interval injection every 168 hours applying 10^6^ DCs per dose during three weeks. Tumor population (*green line*) considering the influence of *T* *G* *F*−*β* in the model and tumor population (*red line*) without influence of *T* *G* *F*−*β* in the model

To discover the effect of the *TGF*−*β* cytokine in the immunotherapy, the model eliminating the influence of the *TGF*−*β* in the immunotherapy were tested. The results show that the effect of *TGF*−*β* in the model increases the tumor cell population. Eliminating the *TGF*−*β* influence, the population of tumor cells is reduced 77.48 *%* and the tumor could be eradicated (Fig. 7).

#### Model sensitivity

In order to know which parameters affect the model outcome a local parametric sensitivity analysis (LPSA) was made [16, 19]. The sensitivity of a single parameter was calculated by comparing the difference between the final number of tumor cells obtained upon changing in ±1 *%* the reference value of the parameter to the final number of tumor cells obtained using the references values of the parameters (Protocol 2, time simulation 1000 hours).

**Fig. 8:**
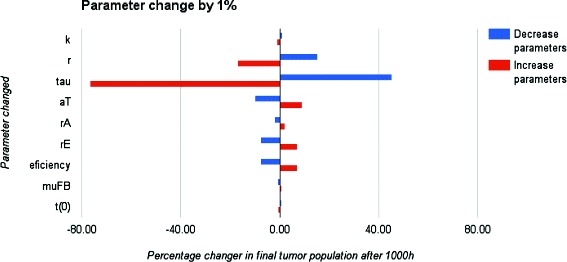
Local Parameters Sensitivity Analysis(LPSA) for the immunotherapy model using the protocol 1 (Table 1)

Figure 8, shows a graphic with the results obtained from the sensitivity analysis. It can be seen that the parameter “ *τ*” has the bigger percentage change in the amount of tumor cells at the end of the model simulation with an interval of change between −76.64 *%* and 45.33 *%*, followed by the tumor growth rate “*r*” and the maximal efficiency of cytotoxic cells “*aT*”.

## Discussion

The mathematical model of 5 DDEs presented is capable of simulating the tumor cells growth with a NRMSE 10 *%* between the results obtained from the model simulation and real immunotherapy data from the biological trials developed by the UNAM researchers. In spite of only taking into account the interactions between the tumor cells, the dendritic cells, the CTLs activated/inactivated and the *TGB*−*β* cytokine the approximation could be considered successful.

The validation of the model allows to test different hypothetical immunotherapy protocol changing the number of infusions or varying the amount of DCs injected.

As a result of these trials, it is observed that if 4 doses of 10^6^ DCs are injected every 96 hours during 3 weeks, the growth of the tumor is less than the current immunotherapy protocol and as a result the mice could live longer. This protocol is viable biologically and experimentally, which could be tested by the research group of UNAM.

By varying the number of DCs injected from the protocol proposed in Table 2, we found that injecting over 10^7^ dendritic cells the tumor is eradicated. Moreover, increasing this amount the DCs *in vitro* is not experimentally viable at the moment for the research group of UNAM due to laboratory limitations.

In case of variation in the percentage of DCs that induce the immune response,the objective is to know whether the number of DCs injected affects the therapy efficiency. The validation model takes into account that not all DCs become effective, around 50 *%* activated and stimulated the reproduction of the CTLs. By performing this experiment, the results show that if more that 80 *%* of DCs arrive at the lymph nodes, the tumor is eradicated. This suggests that if the immunotherapy is improved, making more tumor cells reach the lymph nodes, the tumor could be erradicated.

Currently, some immunotherapies have been including inhibition of “TGF- *β*” cytokine [20], for these reason a hypothetical therapy is suggested in which the effect of this cytokine is eliminated. In this case the results show that the cytokine has a high relevance in the effectiveness of the immunotherapy. A combination of infusion of DCs and the inclusion of inhibitors on the activity of “TGF- *β*” can give better results than the current therapy used.

By performing the sensitivity analysis of the parameter model it was observed that the delay in the time is the most sensitive parameter. Observing in the biological experiment results the main inmunotherapy response is given on the 30th day after injecting DCs into the mice and not on the day when they were injected. The reason for this delay is not clear for the UNAM researchers but it is proposed in the model that the activation and the proliferation of the CTLs was before the CTLs could eliminate the tumor cells this represents the time delay used in the model. The rate of tumor growth is another sensitive parameter and is influenced by the type of cancer (in this case skin melanoma).

On the other hand, the cytotoxity of the CTLs represent the capacity of the CTLs to eliminate the tumor cells and its other sensitivity parameter. One factor more in the sensitivity of the model is the efficiency of the immunotherapy that means the number of dendritic cells that arrive at the lymph nodes to activate and reproduce the CTLs. This efficiency is related with the treatments that the DCs receive before being injected into the mice, and they are not considered in this model for the moment.

For now there is no biological proof to verify the hypothetical treatment. However, the objective of the model is to present a guide for a possible therapy with a greater possibility of success. Future work concerns amplifying the model including more population cells, cytokines or the treatment of DCs before beginning the immunotherapy.

## Conclusions

The model represents a simple view of some mechanisms that occur between the immune system and the immunotherapy with DCs for the melanoma in mice. Even when is only taking into account 5 interactions between the immune cells, tumor cells and one cytokine, the model reproduces in an adequate form the behaviour observed in the experimental biological trials and its validation gives the possibility to prove different hypothetical protocols.

Using the model as a framework the numerical results show that immunotherapy used in the biological experiments could be improve manipulating the number of DCs infused, changing the intervals of injection, changing the effect of “TGF- *β*” cytokine or increasing the percentage of DCs that arrive to the lymph nodes.

Moreover, the model is used to analyse the consequences of manipulating their parameters. It is found that the delay in the time “ *τ*”, the maximal growth rate of the tumor “*r*” and maximal efficiency of cytotoxic cell parameter “ *a*_*T*_” plays a significant role in increasing the effectiveness of the immunotherapy. These results give other hints of how the immunotherapy could be improved.

The the mathematical model can be used as a guide to improve the immunotherapy and thus minimize the costs in time and money to test new protocols. Eventually, the UNAM researchers could have a better possibility of success for a new protocol.

Finally, during the test phase it was observed that the model could be improved by increasing the number of key elements for example the CD4+ helper T lymphocyte response and the B cell response that are related with the production of antibodies with affinity to recognize the antigen melanoma, the secretion of IL-2 cytokine that acts on the activated lymphocytes and stimulates their proliferation, the major histocompatibility complex (MHC) molecules, which are expressed on the surface of the dendritic cells and stimulate the response of the T cells or include a small pre-treatment DCs model in the five DDEs of the model.

## Methods

### Immunotherapy

For the biological experiments, the research group used 10 male mice *C*57*BL*/6 between 6 and 8 weeks of age maintained in the animal facilities of the Medicine Faculty of UNAM. DCs used in the immunotherapy are taken from mice bone marrow and incubated *in vitro* with MG-CSF cytokine to differentiate the DCs and MAGE-AX (25*μ* g/ml) antigen to stimulate the immune response. On the other hand, the formation of melanomas is induced in mice after infusion 6×10^4^ cell *B*16/*F*10 line. Immunotherapy begins in the 10 mice one week latter after the tumor is induced; vaccine protocol uses 10^6^ dendritic cells per dose infused once a week for 3 weeks (Table 1).

The immunotherapy control was made injecting 6×10^4^ tumor melanoma cells in 10 mice (control mice) and allowing the tumor growth without immunotherapy. The efficiency of the biological treatment was measured by comparing tumor diameters, the *IL*−2 and *IL*−10 cytokines modulation, the expression of MHC molecules and the survival rate of mice receiving the inmunotherapy.

The tumor diameter was measured on day 7 once the tumor cells had been injected. Afterwards, they were measure every two days consecutively after day 10 until the mice died or 5 weeks after the first dose of the DCs had been injected.

The results demonstrated that the mice without immunotherapy lived for 28 days, having an average diameter of 4.3*cm*. Injecting the DCs pretreated with *MAGE*−*AX* peptide before the immunotherapy, the outcome showed that some mice lived until the 40*th* day with an average diameter of 1.5*cm*.

Considering the investigation observation of the research group of UNAM that not all of DCs could arrive at the lymph nodes to activate the cytotoxic cells, it takes in consideration that only the 50 *%* of total DCs injected per dose activated the immune response.

### Mathematical model

The proposed model gives a simplified view of the mechanism between the immune system and the immunotherapy developed by the research group at the Medicine Faculty of UNAM, where melanoma in mice is treated with a DCs immunotherapy. The immunotherapy protocol (Table 1) applies three doses of 10^6^ dendritic cell activated with MAGE-AX, infused every 168 hours during four weeks.

The mathematical model is based on the mathematical model proposed by Kronik et al. [21] which describes Glioma and Immune system interactions. They define an ordinary differential equations (ODEs) system to simulate *ex vivo* active alloreactive cytotoxic T-lymphocytes (aCT) treatment to boost the immune response.

DDEs system is proposed taking into account the interactions between the tumor cells population “*T*”, dendritic cells “*D*”, effector and naive cytotoxic T cells “ *C*_*a*_, *C*_*i*_” and the cytokine transforming growth factor *β* “ *F*_*β*_”; this cytokine reduces the efficiency of the immunotherapy, as is shown in [11]. Moreover, the remarks and conclusions made by the research group of UNAM about their treatment are considered as well as that only the activated CTLs can eliminate the tumor cells and these cells produce the transforming growth factor *β* (TGF- *β*). A delay in the time, “ *τ*” is included in the model to describe the time of activation of dendritic cells.

Some parameters are obtained from the bibliography, and others are calculated using the model of DDEs, adjusting their values to biological treatments results, trying to minimize the difference of the numerical data with the experimental data, see the supplementary material for a complete explanation.

### Mathematical equation

#### Tumor cells, “*T*”

To study the growth of tumor cells population “*T*”, Gompertz growth law was considered (it provides a better fit to the experimental data than the logistic law) first term on the right hand side (RHS) of Eq. 1, where the parameter “*r*” represents the maximal tumor growth rate and the parameter “*K*” its carrying capacity (the limit of the maximum population size of the tumor cells that the environment can sustain). Notice that the number of cells in a mouse is significantly lower than “K”, we consider Gompertz growth if *T*<1.6×10^10^ and the experiments stop if *T*=1.6×10^10^. It is worth to mention, that at if *T*=1.6×^10^ the mice died.

For the elimination of tumor cells by the CTLs, “ *C*_*a*_” (second term on the RHS of Eq.1), it is assumed that both “*T*” and “ *C*_*a*_” are proportional, the maximal efficiency rate of CTLs is denoted by “ *a*_*T*_”.

The influence of TGF- *β* cytokine, “ *F*_*β*_”, is also considered. It produces a reduction of immunotherapy efficiency and it is assumed to be an immunosuppresive factor of CTLS activity, Michaelis-Menten form represented by the factor $a_{T,\beta }+\frac {e_{T,\beta }(1-a_{T,\beta })}{e_{T,\beta }+F_{\beta }}$ is used for that purpose. Where “ *e*_*T*,*β*_” is the Michaelis-Menten constant and the maximal reduction effect of TGF- *β* on CTLs efficiency is “ *a*_*T*,*β*_”. This term is similar to that used by Kronik et al. [21].

(1) $$ {\small{\begin{aligned} \frac{dT}{dt} =rT\ln\left(\frac{K}{T}\right)-a_{T} \cdot C_{a}\cdot T\cdot\left(a_{T,B}+\frac{e_{T,B}(1-a_{T,B})}{e_{T,B}+F_{\beta}}\right) \end{aligned}}}  $$

#### Dendritic cells, “*D*”

Equation 2, describes the population of the dendritic cells dynamics, “*D*”. The initial number of dendritic cells is consider, *d*_0_ which decay at constant rate *μ*_*D*_, then, for *n*<168 the number of dendritic cells is given by

$$ D(t)=0 \qquad \text{if t} < 168 $$

at *t*_*n*_=*n*·168 for *n*=1⋯3, 10^6^ dendritic cells are injected into the mouse, so, the number of dendritic cells of the immunotherapy is given by

$$\begin{array}{@{}rcl@{}} D(t):= \left\{ \begin{array}{lcl} 0, & & \text{if} \quad t <168 \\ [3mm] 10^{6} e^{-\mu_{D}(t-168) }\cdot ef, & & \text{if} \quad 168 \leq t <168 \cdot 2 \\ [3mm] 10^{6} \left(e^{-\mu_{D}(t-168)} + e^{-\mu_{D}(t-168 \cdot 2)} \right)\cdot ef, & & \text{if} \quad 168 \cdot 2 \leq t <168 \cdot 3 \\ [3mm] 10^{6} \left(e^{-\mu_{D}(t-168)} + e^{-\mu_{D}(t-168 \cdot 2) }+ e^{-\mu_{D}(t-168 \cdot 3)} \right)\cdot ef, & & \text{if} \quad t \geq 168 \cdot 3. \end{array} \right. \end{array} $$

Where, *ef* represents the percentage of the DCs which arrive the lymph nodes to begin the immune response. Notice that the evolution of dendritic cells inside the intervals (168(*n*−1),168*n*) for *n*=1⋯3 is given by

(2) $$ \frac{dD}{dt}= -\mu_{D} D, \qquad t \in (168(n-1), 168 n)\ \text{for}\ \, n=1 \cdots 3  $$

#### Cytotoxic T lymphocyte activated, “ *C*_*a*_”

The dynamic of CTLs activated, “ *C*_*a*_”, is described in Eq. 3 considering the activation of naive *CD*8^+^, expansion of the CTLs activated and a natural death of CTLs activated.

The first term on the right side of the equation represents the cytotoxic cells activation. It is considered that the contact between the DCs and the inactive cytotoxic cells produce the activation of these. It is proposed that the activation is made before the death of the tumor cells and gives a rate *r*_*a*_, The encounter between the inactivated cytotoxic cells and DCs occurs at the time of *t*−*τ*. On the other hand, the survival probability of the inactive cytotoxic cells during the delay time is considered by the term $e^{- \mu _{c_{i}} \tau }$ of the equation 3. It is assume that the effect of the dendritic cells in the activation of the CTLs follows a Michaelis-Menten saturation dynamic with a constant of “ *θ*_*D*_”.

The second term on the right side of the equation represents the expansion of the activated cytotoxic cells. This expansion shows a rate of *r*_*e*_, given for the contact between the DCs and the activated cytotoxic cells (described as a saturation function of type Michaelis-Menten). In the same way as the cytotoxic activation cells, the expansion is given before the death of the tumor cells and occurs at the contact time of *t*−*τ* between the DCs and the activated cytotoxic cells. The probability of the activated cytotoxic cell survival is consider during the time delay in term $e^{- \mu _{c_{a}} \tau }$ of the equation 3.

The natural death of the activated cytotoxic cells is included in the last term of the equation 3, with a constant death rate “$\mu _{C_{a}}$”.

(3) $$\begin{array}{@{}rcl@{}} \frac{{dC}_{a}}{dt} &=& r_{a}\cdot e^{(-\mu_{C_{i}}\tau)}\cdot C_{i}(t-\tau) \cdot \left(\frac{D(t-\tau)}{D(t-\tau) + \theta_{D}}\right) \\ &&+ r_{e}\cdot e^{(-\mu_{C_{a}}\tau)}\cdot D(t-\tau) \cdot\left(\frac{C_{a}(t-\tau)}{C_{a}(t-\tau)+\theta_{a}}\right) - \mu_{C_{a}} C_{a}. \end{array} $$

#### Cytotoxic T lymphocyte inactivated, “ *C*_*i*_”

Equation 4, express the inactivated cytotoxic cells dynamic of the population “ *C*_*i*_”. The first term on the right hand side of the equation is equal to the first term of the equation 3 and represents the decrease of the inactivated cytotoxic cells which will be activated. The contact between these populations occur in the time *t*−*τ*.

Due to the mice immune system not presenting any response before being injected by immunotherapy, it is not considered a term that produces new inactivated cytotoxic cells. The model only takes into account a base amount of inactivate cytotoxic cells that is introduced into the model as an initial condition (see Additional file 1).

The effect of dendritic cells in the CTLs activation is supposed following a Michaelis-Menten dynamic and the dependence of “ *C*_*i*_” efficiency on “*D*” population is represented by Michaelis constant “ *θ*_*D*_”.

A constant death rate “$\mu _{C_{i}}$” is assumed for the cytotoxic inactivated cells.

(4) $$ \frac{{dC}_{i}}{dt} = - r_{a}\cdot e^{(-\mu_{C_{i}}\tau)}\cdot C_{i}(t-\tau) \cdot \left(\frac{D(t-\tau)}{D(t-\tau)+\theta_{D}}\right) -\mu_{C_{i}} C_{i}  $$

#### Transforming growth factor, “ *F*_*β*_”

Equation 5, describes the cytokine dynamic of TGF- *β*, “ *F*_*β*_”. The term “ *a*_*T*,*β*_” represents the production rate per tumor cell. It is proportional to the number of tumor cells, “*T*”. The last term of the equation is a natural degradation rate “ *μ*_*β*_” of the TGF- *β*.

(5) $$ \frac{{dF}_{\beta}}{dt} = a_{T,\beta}T-\mu_{\beta} F_{\beta}  $$

### Computer simulation

The model is implemented using a 4th order Runge Kutta method with the integration step of 1 hour [22, 23]. Some parameters had been obtained from literature and others had been calculated using the model and based on *in vivo* experimental results of immunotherapy developed by the research group. The additional material presents the references used to obtain the model parameters (see Additional file 1).

The experiment data is used to calculate the value of maximal growth rate of tumor, “*r*” and the maximal population size of the tumor cells that the environment can sustain “*K*” using least square method and setting an initial population size of tumor cells at 6∗10^4^ according the immunotherapy protocol applied by the research group of UNAM (Table 1).

The maximal efficiency of cytotoxic cells “ *a*_*T*_”, the activation rate of CTLs “ *r*_*a*_”, the expansion rate of activated CTLs “ *r*_*e*_” and the delay in the time “ *τ*” are calculated after an exhaustive search minimizing the difference between the numerical results and the experimental data, the additional material shows more detail (see Additional file 1).

## Additional file

Additional file 1
**Description of parameters used in the mathematical model.**

## Abbreviations

DCs: Dendritic cells
CTLs: cytotoxic T lymphocytes
TGF- *β*: Tumor growth factor *β*
MAGE-AX: Melanoma-associate antigen AX
MG-CSF: Granulocyte macrophage colony stimulating factor
NRSME: Normalized root mean square error
LPSA: Local parameter sensitivity analysis
