# Supplementary material for: Enhancing dendritic cell immunotherapy for melanoma using a simple mathematical model
Source: Theor Biol Med Model. 2015 Jun 9;12:11. doi: 10.1186/s12976-015-0007-0 (PMC4469008; doi:10.1186/s12976-015-0007-0)
Supplement: Additional file 1 — Description of parameters used in the mathematical model. [file 12976_2015_7_MOESM1_ESM.pdf]

Additional Material

Parameters values

The mathematical model was focused on one of the biological experiments performed by Gabriela et. al [1]. which made a DCs pre-treatment with MAGE-AX before being injected into the mice. The maximal growth rate "r" and the tumor carrying capacity "K" were calculated using a least square method to adjust a Gompertz function in order determine the melanoma growth in the mice under the proper biological experimental conditions without immunotherapy.

Maximal growth rate of tumor, "r". For its estimation is taken into account the estimation of average tumor diameters, measured by the UNAM researchers (the 7th day after melanoma cell induction and every two days after the 10th day for a period of 700 hours) in control mice immunotherapy; a spherical shape is assumed for the measured tumors and the average population is obtained on tumor cells (considering the size of melanoma tumor cell equal to $17.4 \pm 0.21\mu$ m [2]).

Using the least squares method a growth rate of blue 0.0010602 with an error of 4.95% was obtained.

Tumor carrying capacity, "K".The maximum population sizes of tumor cells can be limited by the environment and represented by the tumor carrying capacity and is calculated using the same adjustment of the maximal growth rate of tumor "r" parameter. The search for the value of "K" is carried in a range between 10^{15} and 10^{20} and the value determined was $6.75 * 10^{15}$ cells.

In another vein, observing the biological results, the mice died when the tumor reached a diameter of 4.3 cm (approximately $1.6 * 10^{10}$ tumor cells); for that reason this population was taken as a limit of the population tumor cells in the simulation and was considered constant from that moment.

Natural death rate of DCs, "μ_D". De Pillis et al. [3] Use the studies of Ludewing et al. [4] to determinate the value of "μ_D" in $0.009625h^{-1}$.

Death rate of activated CTLs in tumor compartment, "μ_{C_a}" The value "μ_{C_a}" is calculated at $0.01925h^{-1}$ by De Pillis et al. [3].

Threshold in DC density for half-maximal proliferation rate of CTLs, "θ_D". Set on 212 cells and determinated by De Pillis et al. [3].

Threshold in "C_a" density for half-maximal proliferation rate of CTLs, "θ_A". Found in a interval search between [1, 300] and calculated at 10.

The proposed model and the experimental data of inmunotherapy in mice with melanoma is used to find the "a_T", "τ", "r_E" and "r_A" parameters performing an exhaustive research and minimizing method; the minimum mean square error value is taken as a reference value to determine the optimum value of the parameters. The minimum mean square error is a result of a comparison between the tumor cells population of the biological experiments and the tumor cell population generated by the model. The parameters value found is:

Maximal efficiency of cytotoxic cells, "a_T". This parameter represents the efficiency at which the cytotoxic cells kill the tumor cells. Its estimation is carried out through an exhaustive search in an interval search between 10^{-13} and 10^3 the value obtained is $6 * 10^{-11} h^{-1}$.

Activation rate of CTLs, "r_a". A interval search between 0.00001 and 100 is used to find the most optimal, estimated at $61h^{-1}$. The data used in the models proposed by Ludewing et al. [4] and Handel et al. [5] is considered to determine the interval search.

Expansion rate of activated CTL, "r_e". The value of "r_e" parameter is set at $6500h^{-1}$ which is the result of the estimation using an exhaustive search in an interval search between $10h^{-1}$ and $7000h^{-1}$

Delay in the time, "τ". Indicates the time when the therapy is becoming effective, considering that this is effective between 100 and 300 hours after injecting the first immunotherapy, and taking into account the data of immunotherapy biological tests the value of "τ" is determined in 265 hours by an exhaustive search.

The parameters listed below are taken for the Kronik et al. [6] study.

Maximal reduction effect of "$TGF-\beta$" on CTLS efficiency, "$a_{T,\beta}$". Using the reports about the CTL efficacy the value determinated by Kronik et al. is $0.69h^{-1}$.

Dependence of CTLs efficiency on $TGF-\beta$, "$e_{T,\beta}$". Estimated at $10^4 pg$.

Death rate of inactivated CTLs, "μ_{C_i}" . The model uses the CTLs half life estimated by Kronik et al.[6] in $0.007h^{-1}$.

Production rate of $TGF-\beta$ by a single tumor cell, "$r_{T,\beta}$". Set at $5.75 * 10^{-6}$ $pg(cell * h)^{-1}$.

Degradation rate $TGF-\beta$, "μF_β". Is found in a interval search between $1h^{-1}$ and $10h^{-1}$, set at $2.7h^{-1}$.

Simulation of the mathematical model trajectories, Protocol 1 and 12

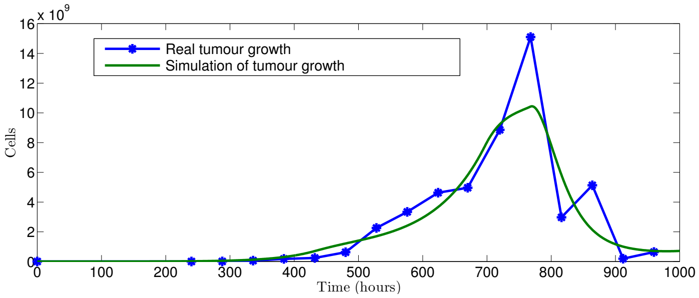

Figure 1: Simulation cytotoxic cell growth with the Protocol 1. Simulation for 1000 hours set to $T(0) = 6 \times 10^4$, $r = 0.00106h^{-1}$ and $K = 6.754 \times 10^{15} cells$.

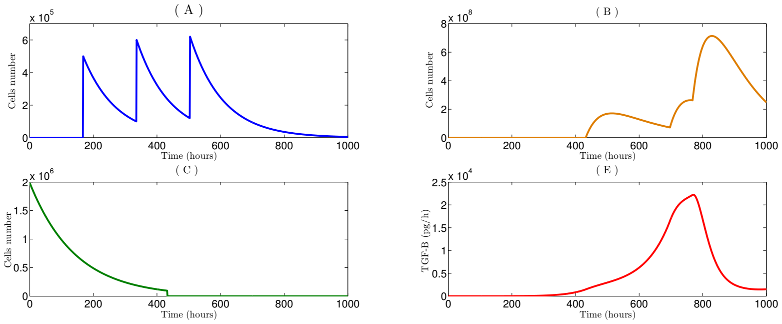

Figure 2: Simulation of dendritic cells dynamic (A),CTLs activated dynamic (B),CTLs inactivated dynamic (C) and TGF-B dynamic (D) with the Protocol 1. Simulation for 1000 hours set to $T(0) = 6 \times 10^4$, $r = 0.00106h^{-1}$ and $K = 6.754 \times 10^{15} cells$.

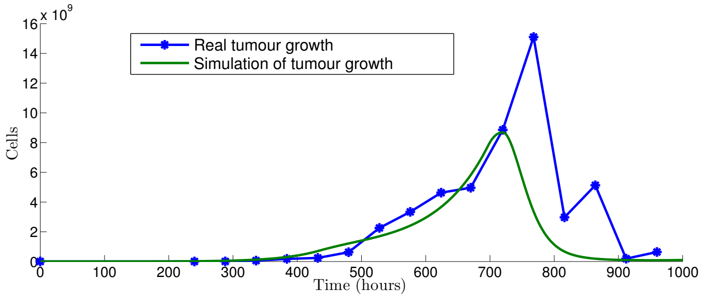

Figure 3: Simulation cytotoxic cell growth with the Protocol 2. Simulation for 1000 hours set to $T(0) = 6 \times 10^4$, $r = 0.00106h^{-1}$ and $K = 6.754 \times 10^{15} cells$.

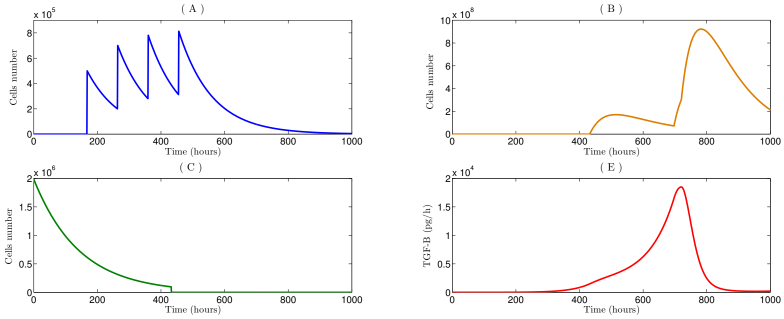

Figure 4: Simulation of dendritic cells dynamic (A),CTLs activated dynamic (B),CTLs inactivated dynamic (C) and TGF-B dynamic (D) with the Protocol 2. Simulation for 1000 hours set to $T(0) = 6 \times 10^4$, $r = 0.00106h^{-1}$ and $K = 6.754 \times 10^{15} cells$.

References

[1] Piñon-Zarte, G., Herrera-Enríque M. A., Hernandez-Téllez, B., Jarquín-Yáñes, K., Castell-Rodríguez, A. E. GK-1 improves the immune response induced by bone marrow dendritic cells loaded with MAGE-AX in mice with melanoma. Journal of Immunology Research. 2014;**2014**:1-12.

[2] Ochalek, T., Nordt, F.J., Tulberg, K. Correlation between cell deformability and metastatic potential in B16-F1 melanoma cell variants. Cancer Research. 1988; **48**: 5124-5128.

[3] de Pillis, L.G., Gallegos, A., Radunskaya, A.E. A model of dendritic cell therapy for melanoma. Frontier in oncology. 2013; **3**:1-12.

[4] Ludewing, B., Krebs, P., Junt, T. Determining control parameters for dendritic cell-cytotoxic T lymphocyte interaction. Eur. J. Immunol.2004;**34**:2407-2418.

[5] Andreas Handel and Rustom Antia. A Simple Mathematical Model Helps To Explain the Immunodominance of CD8 T Cells in Influenza A Virus Infection. Journal Of Virology. 2008; doi:10.1128/JVI.00653-08.

[6] Kronik, N., Kogan, Y., Vainstein, V., Agur, Z. Improving alloreactive CTL immunotherapy for malignant gliomas using a simulation model of their interactive dynamics. Cancer Immunol Immunother. 2008; **57**:425-439.

[7] Thomas, D. A., Massagué. J. Tgf-β directly targets cytotoxic T cell functions during tumor evasion of immune surveillance. Cancer Cell. 2005;**8**:369-380.

[8] Perterson, P. K., Chao, C. C., Thielen, K., Shaskan, E. G. Glioblastoma, transforming growth factor-beta, and Candida meningitis: a potential link. Am J. Med. 1992; **92**:262-264.

[9] Coffey, R. J., Kost, L. J., Lyons R. M., Moses, H. J., LaRusso, N. F. Hepatic Processing of Transforming Growth Factor B in rat uptake, metabolism, and biliary excretion. J. Clin. Invest. 1987; **80**:750-757.

[10] Duriancil, D. M., Katheleen A.H. The Identification and Enumeration of dendritic cell populations from individual mouse spleen and Peyer's patches using flow cytometric analysis. Cytometry Part A. 2009;**75**: 951-959.

Table 1: Parameter used in the current model

Parameter	Description	Estimated value	Reference
r	Maximal growth rate of tumor	0.001060289 h^{-1}	Fit to Gompertz function
K	Tumor carrying capacity	6.75×10^{15} cells	Fit to Gompertz function
a_T	Maximal efficiency of citotoxic cells	$6\times10^{-11}\ h^{-1}$	Estimated
$a_{T,\beta}$	Maximal reduction effect of $TGF-\beta$ on CTLS efficiency	$0.69 none$	Base on data of Kronik [6] and Thomas [7]
$e_{T,\beta}$	Dependence of CTLs efficiency of $TGF-\beta$	$10^4 pg$	Base on data of Peterson [8] and Kronik [6]
μ_D	Natural death rate of DCs	$0.009625Xh^{-1}$	Ludewing [4] and De Pillis [3]
r_a	Activation rate of CTLs by DC	$61h^{-1}$	Estimated
θ_D	Threshold in DC density in the spleen for half-maximal proliferation rate of CTLs	$212 cells$	Based on Ludewing [4]
r_e	Expansion rate of activated CTL	$6500h^{-1}$	Estimated
μ_{C_a}	Dead rate of activated CTLs in tumor compartment	$0.01925h^{-1}$	De Pillis [3]
θ_A	Threshold in C_a density for half-maximal proliferation rate of CTLs	$10 cells$	*Ad hoc* fixed value
μ_{C_i}	Dead rate of inactivated CTLs in tumor compartment	$0.007h^{-1}$	*Ad hoc* fixed value base on Kronik [6]
$r_{T,\beta}$	Production rate of $TGF-\beta$ by a single tumor cell	$5.75 * 10^{-6} pg(cell*h)^{-1}$	Base on data of Peterson [8] and Kronik [6]
μ_β	Degradation rate of $TGF-\beta$ by a single tumor cell	$2.7h^{-1}$	Fit to Coffey [9] and Kronik [6]
τ	Delay in the time to be effective the immunotherapy	$265h$	Estimated
$DCsize$	Diameter of Dendritic Cell	1.74μ	Ochalek [2]

Table 2: Initial condition of the model

Model variable	Value	Interval in the time	Reference
$T(t)$	6×10^4	$[-\tau, 0]$	Gabriela et. al. [1]
$D(t)$	0	$[-\tau, 0]$	
$C_a(t)$	0	$[-\tau, 0]$	
$C_i(t)$	2×10^6	$[-\tau, 0]$	Duriacil et. al [10]
F_β	0	$[-\tau, 0]$	
